# Effects of methylphenidate and physiotherapeutic treatment on graphomotor movements in children with ADHD

**DOI:** 10.1007/s00787-023-02144-5

**Published:** 2023-01-23

**Authors:** Josefine Rothe, Fabian A. Kattlun, Jeanne Kaufmann, Anne Uhlmann, Sina Wanderer, Annet Bluschke, Christian Beste, Veit Roessner

**Affiliations:** Department of Child and Adolescent Psychiatry and Psychotherapy, Technische Universität Dresden, Dresden, Germany

**Keywords:** Developmental coordination disorder, Motor problems, Handwriting, Digitizing tablet

## Abstract

In addition to the core symptoms defining ADHD, affected children often experience motor problems; in particular, graphomotor movements including handwriting are affected. However, in clinical settings, there is little emphasis on standardized and objective diagnosing and treatment of those difficulties. The present study investigated for the first time the effects of methylphenidate as well as physiotherapeutic treatment on objectively assessed graphomotor movements compared to a control condition, i.e. parental psychoeducation, in 58 children (mean age: 9.52 ± 1.91 years) newly diagnosed with ADHD in an outpatient clinic for child and adolescent psychiatry. Families were invited to join one of the treatment groups. Before and after 8 weeks of treatment, children performed six different tasks on a digitizing tablet which allowed the objective analysis of three important kinematic parameters of graphomotor movements (fluency, velocity, and pen pressure) in different levels of visual control and automation. Graphomotor movement fluency and velocity improves over time across the groups, especially in tasks with eyes closed. We did not find clear evidence for beneficial effects of methylphenidate or physiotherapeutic treatment on children’s overall graphomotor movements suggesting that treatments need to be better tailored towards specific and individual deficits in graphomotor movements.

**Supplementary Information:**

The online version contains supplementary material available at 10.1007/s00787-023-02144-5.

## Introduction

Attention-deficit/hyperactivity disorder (ADHD) is a neurodevelopmental disorder characterized by the core symptoms inattention, impulsivity and hyperactivity that occurs in multiple contexts and cannot be attributed to another diagnosis [1]. Prevalence estimates for school age children range from 5.9 to 11.4%, making it the most common disorder in children and adolescents [2]. Beside its core symptoms, 30–50% of children with ADHD display motor problems that may culminate in a coexisting developmental coordination disorder (DCD) [3, 4]. Relative to their age, children with DCD show substantial impairments in various aspects of motor coordination (e.g. fine and gross motor skills). Often, graphomotor movements (GM) (e.g. handwriting) are also affected [5–8], which further impacts academic achievement [1, 9]. Physiotherapeutic treatment is commonly prescribed to improve motor problems.

Children spend a substantial amount of time (30–60%) of their school day performing fine motor tasks, with 17–37% accounted for by paper–pencil tasks, including handwriting with 3–18% [10, 11]. It is therefore undeniable that impairments in GM can interfere not only with academic achievement in particular (e.g. fluency in handwriting letters predicts written expression), but also with children’s development in general (e.g. lower self-esteem, more frustration over time-consuming writing) [12, 13]. Considering the implications of impaired fine motor skills including GM on children’s academic achievement, social participation and mental health which often persists during adolescence and adulthood [12, 13], there is a need for evidence about specific treatment effects on fine motor skills including GM. There are reports about interventions to tackle general motor coordination problems, however, GM are barely investigated. Schoemaker et al. [14] investigated the effects of a three month physiotherapeutic treatment on motor coordination in children with DCD compared to controls. Fine motor skills, which were measured by pencil control (line tracing task), did not improve.

**Fig. 1:**
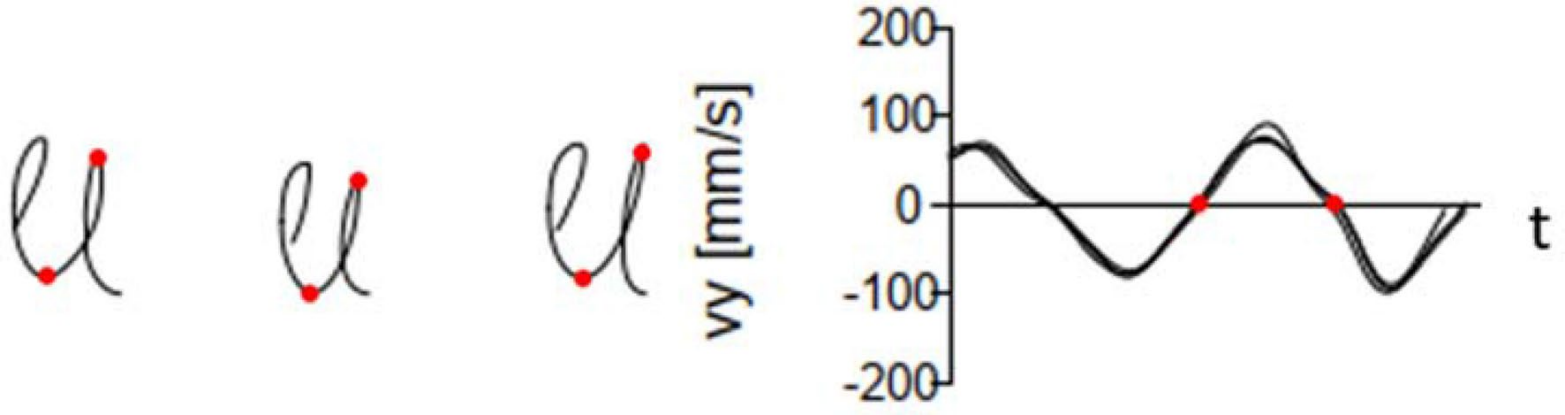
Example of a velocity profile in automated writing double-loops three times. On the left side, the three times written double loops are shown. On the right side, the velocity profile for writing the three double loops is arranged one above the other. The velocity during the upstroke is displayed above zero, while the velocity during the downstroke is displayed below zero. Thus, each stroke starts and ends with the corresponding velocity at zero. The red dots mark the middle upstroke. vy = velocity measured in mm/s; t = time in seconds

Digital technology allows ongoing improvements in the objectification and quantification of GM through the continuous registration of kinematic parameters in standardised tasks. Breaking down the GM into strokes allows the analysis of numerous kinematic variables. For example, the position coordinates of pen movements are decomposed into segments, so-called strokes (see Fig. 1). The two semicircles within a full circle—upstroke and downstroke—are evaluated as two separate strokes. A stroke segment starts and ends when the velocity in the direction of the *y*-axis passes through zero. The fluency of GM can be measured by the **N**umber of **I**nversions in **V**elocity per stroke (NIV). Velocity profiles of experienced writers follow a distinct pattern. They are bell-shaped and symmetric which means that for every stroke there is only one inversion in velocity (NIV = 1) [15, 16]. There are two more variables highly relevant for the analysis of GM: the stroke frequency per second as a measure of GM velocity and axial pen pressure, the exerted force of the pen on the surface. Even though experienced observers can visually detect differences in writing velocity and pen pressure, it is not comparable with the objectification and quantification through a continuous digital registration of kinematic parameters. Furthermore, handwritten graphemes might look neat and tidy to the investigator's eye, but might still be characterised by many inversions in the velocity profile (i.e. a dysfluent writing with many stops and starts within one stroke).

Over the last years, some studies on GM in children with ADHD utilizing a digitizing tablet have been published [17–22]. Some of them demonstrated that children with ADHD write and draw faster than control children which is associated with a more inaccurate, ‘messy’ writing product [17, 18, 20]. Taken together, high velocity and diminished accuracy can be interpreted as a speed-accuracy trade-off of GM in children with ADHD.

Furthermore, there is evidence that pen pressure is increased in children with ADHD [17, 19]. Adi-Japha et al. [17] argued that children with ADHD increase the mean pen pressure in order to gain better control when writing. However, increased pen pressure had no effect on fluency.

While some of the above mentioned studies investigated GM in everyday handwriting tasks (i.e. writing a sentence as well as multiple-segments consecutively from left to right), other studies used tasks that rather tap into basic drawing movements (e.g. drawing of superimposed circles). Particularly, basic drawing movements can further be investigated under various degrees of visual control and automation, i.e. with open or closed eyes (visual control) and using the dominant or non-dominant hand (automation). In recent literature, visual control has been reported to substantially affect GM in everyday handwriting tasks (e.g. [22]).

As handwriting legibility of children with ADHD is generally diminished, some research has focused on the effects of methylphenidate treatment on GM [20, 21]. For example, Brossard-Racine et al. [23] examined changes in handwriting performance of children newly diagnosed with ADHD prior to and 3 months following use of a stimulant medication. An occupational therapist scored the legibility of the handwriting on a scale of 0–100%. Handwriting legibility improved significantly 3 months after initiation of medication, but most children continued to show handwriting legibility difficulties after initiation of medication. Furthermore, another study demonstrated that legibility scores of handwriting of children with ADHD on methylphenidate are not significantly different from those of controls, whereas their handwriting legibility after methylphenidate withdrawal is significantly worse than that of controls [20]. Again, it has to be noted, that legibility was rated by examiners using 5-point scales ranging from excellent to poor and is, therefore, not as objective as digital registration of GM. Strikingly, it is also shown that children with ADHD had less fluent GM in sentence writing on methylphenidate compared to the same children after methylphenidate withdrawal (time to the last medication was approximately 10–12 h) [20, 21]. The authors conclude that MPH enables children to focus their attention on conscious control of handwriting, which in turn leads to increased legibility but decreased automation.

**Fig. 2:**
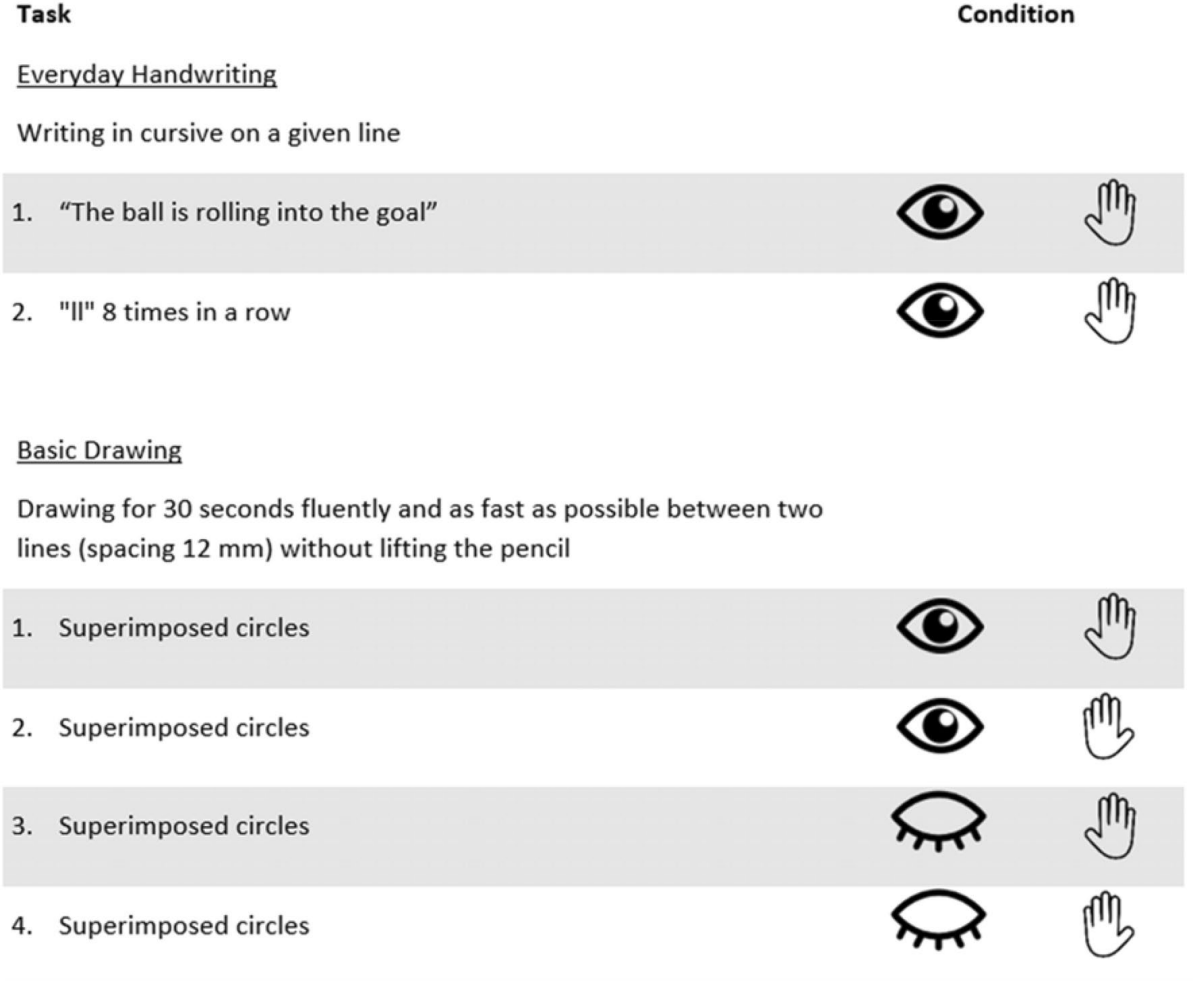
Description of the six GM tasks and related conditions

In sum, there is evidence of faster and more forceful but less accurate GM in children with ADHD, while evidence about specific treatment effects on fine motor skills including GM is scarce. Thus, the goal of the current study is to compare for the first time the influence of three conventional treatments (methylphenidate treatment vs. physiotherapeutic treatment vs. parental psychoeducation as control treatment) on objectively assessed GM in children with ADHD in a pre–post design. A treatment interval of 8 weeks, which is common in the clinical setting, was used for the pre-post design. GM parameters (fluency, velocity and pen pressure) were investigated in everyday handwriting tasks (i.e. writing a sentence as well as multiple-segments consecutively from left to right) and tasks that rather tap into basic drawing movements (e.g. drawing of superimposed circles). Basic drawing movements were assessed under four conditions (eyes open or closed; dominant or non-dominant hand; see Fig. 2). Across all treatment-groups and tasks we assume more fluency, velocity and pen pressure of the dominant hand compared to the non-dominant hand before and after week 8 of treatment. Drawing on earlier findings we assume that children on methylphenidate focus on conscious visual control of handwriting in tasks performed with eyes open (i.e. in both everyday handwriting tasks and in two of the four basic drawing tasks) and thus write less fluently (i.e., NIV is higher), slower, and more forcefully (higher pen pressure) than before starting methylphenidate treatment [20, 21]. In the tasks in which children have to close their eyes (two of the four basic drawing tasks) the focus on conscious visual control of drawing is diminished, and therefore, expectations in task performance are not the same as in the closed eyes conditions. There is evidence that visual feedback processing is impaired in children with handwriting difficulties and thus, visual control during GM may have a more disruptive effect on GM parameters such as fluency and velocity, which does not apply in healthy controls [24]. In addition, kinesthetic sense (senses of position and movement of the body) seems to be associated with better handwriting legibility in general [25]. We do not expect methylphenidate to promote kinesthetic sense and thus improve fluency or speed in the closed-eye conditions. Therefore we expect that GM are similarly fluent, fast and forceful under methylphenidate than before treatment. Assuming that the physiotherapeutic treatment of the present study (specifically designed to treat and train children’s fine and gross motor skills as well as tactile/vestibular/proprioceptive perception) leads to improvements in gross and fine motor skills [26–29], we expect that GM in both everyday handwriting tasks and in the four basic drawing tasks (two open-eye and two closed-eye drawing tasks) will be more fluent (i.e., lower NIV values) and less forceful (lower pen pressure) but slower (i.e., lower frequencies) due to a trade-off between velocity and legibility [30] after week 8 of treatment than before treatment. As the parental education group did not involve direct treatment of the children, it was treated as a control group for which we do not expect any treatment effects.

## Methods

### Subjects and treatment options

Fifty-eight children (48 boys, ten girls; mean age: 9.52 ± 1.91 years) newly diagnosed with ADHD by child and adolescent psychiatrists/psychotherapists of our out-patient clinic for child and adolescent psychiatry completed all six GM tasks. Diagnosis of ADHD was based on clinical assessments (ICD-10), interviews with the children and their parents, and questionnaire outcomes. The multi-faceted diagnostic process also entailed an IQ test, attention testing, medical check-up (blood sampling, a clinical EEG) as well as examinations of sight and hearing to exclude underlying visual and auditory problems. Physiotherapists additionally conducted a motor assessment with children (M-ABC 2, Movement Assessment Battery for Children, 2nd Version [31]) to identify children with below average motor skills (PR ≤ 16). After the ADHD diagnosis was confirmed and there was an indication for treatment, families were informed about three different treatment options and the possibility to participate in the present research study. In accordance with the therapist’s recommendations, families were free to join one of the three treatment groups. Children with neurological disorders, head injury, metabolic disorder or below average intelligence (IQ < 85) were excluded.

*Methylphenidate medication treatment-group* (*MPH):* children (*n* = 18) started medication (dose: 10–40 mg) under supervision of a physician according to international pharmaceutical guidelines within the usual clinical setting. For eight weeks the physician examined the child weekly.

*Physiotherapeutic treatment-group (PHY):* children (*n* = 17) attended appointments with a physiotherapist twice a week over eight weeks. Physiotherapeutic treatment was specifically designed to treat and train children’s fine and gross motor skills utilizing different tasks. These tasks involved stimulation of the physical senses (i.e. tactile/vestibular/proprioceptive perception), creating balance in tonus relation (e.g. by strengthening weakened parts of the muscular system) and training of specific fine and gross motor skills (e.g. responsiveness, orientation and balance). Treatment was always personalized to the subject’s specific weaknesses and needs. In four participants who had already started medication before the start of physiotherapeutic treatment, medication remained stable.

*Parental psychoeducation treatment-group (PP):* during this intervention, parents (of *n* = 23 children) met in eight weekly sessions under therapeutic supervision to receive information about adequate parenting methods for children with ADHD symptomatology as well as to talk about daily struggles and share experiences with other affected families. In nine participants who had already started medication before the start of parental psychoeducation, medication remained stable.

The study was approved by the local ethics department. Written informed consent was provided by the children and their parents before treatment start.

### Procedure and materials

Participants completed two test sessions, one before treatment commenced and one after eight weeks of treatment. Participants in the MPH group were first tested just before starting treatment with methylphenidate and a second time on methylphenidate after week 8 of treatment. GM were registered by a digitizing tablet (WACOM INTUOS4, Wacom, Neuss, Germany) with a specific pen containing no ink refill. The current position of the pen on the tablet, velocity, acceleration and axial pen pressure were measured continuously during the writing process. Localization of the tip of the pen was possible with an accuracy of 0.25 mm in both directions (*x/y*) at a frequency of 200 Hz. For data processing, a commercial software for the analysis of GM was utilized (CSWin [32]. Writing conditions were nearly natural because the tablet was constructed to resemble a common desk pad. Children were not instructed to write neatly, accurately, or legibly.

The procedure and each task was explained verbally to the child by the experimenter, and the child was given a single sheet of paper with the instructions for each task lying on the tablet surface to write on it. Children were not able to see the recordings of their drawings/writings in real time.

GM testing comprised six tasks (see Fig. 2). Two tasks involved *everyday handwriting*. The remaining four tasks involved *basic drawing* movements in different levels of visual control (eyes open or closed) and automation (dominant or non-dominant hand).

### Kinematic parameters of GM

#### Fluency (NIV)

The degree of automation in GM is investigated through fluency profiles, measured by the Number of Inversions in Velocity per stroke (NIV). Automated GM are characterized by smooth and single peaked velocity. Thus a NIV of 1 reflects the highest possible degree of fluency, while higher NIV values indicate less fluent GM [15, 16]. For healthy children and adolescents aged six to eighteen the mean NIV ranges between 1.12 and 1.55 when drawing circles and between 1.38 and 2.50 when writing a sentence (aged eight to eighteen) [33].

#### Velocity

The velocity of GM is measured by number of upward and downward movements in 1 s. In adults, the mean amount of velocity is about 5 Hz [34]. For healthy children and adolescents the mean velocity ranges between 2.62 and 3.61 Hz when drawing circles and between 1.69 and 3.06 Hz when writing a sentence [33].

#### Pen pressure

The pressure exerted by the pen on the pad is continuously measured by a pressure transducer. In adults, the mean amount of pen pressure varies between 1 and 1.5 Newton [34]. For healthy children and adolescents the mean pressure ranges between 1.86 and 2.81 Newton when drawing circles and between 1.87 and 2.49 Newton when writing a sentence [33].

### Statistics

Data of three subjects were excluded from analysis since GM data of two subjects were significant outliers (> 3 standard deviations) and data of one subject was incomplete (remaining sample, *N* = 55). Differences between the groups in sex and below average motor skills were analysed by Pearson’s chi-squared tests. Group differences for age and the means of fluency, velocity and pen pressure were analysed by Kruskal–Wallis tests. Due to the skewed distribution in the 6 different tasks, Box–Cox transformations [35] were applied for fluency with the following lambdas (task 1, 2, 5 and 6 *λ* = − 0.90; task 3 and 4 *λ* = − 0.50) and with lambda 0.50 (square rout) for velocity and pen pressure in all tasks to achieve normal distribution of the data and were used to run mixed ANOVAs. Means and standard deviations of transformed values are shown in Tables 1–3 of supplementary materials. Boxplots of untransformed values are shown, separately for treatment-group and time, in Figs. 2–4 of supplementary materials.

Data of the two *everyday handwriting* tasks were analysed by 2 × 2 × 3 design mixed ANOVAs with the within-subject factors time (pre, post) and task (sentence, double-loops), and the between-subject factor treatment-group (MPH, PHY, PP) separately for fluency, velocity and pen pressure as dependent variables. Post hoc tests for pairwise comparisons of estimated marginal means using Bonferroni correction were run.

For the sake of better comprehensibility, we have chosen to use the term four *basic drawing tasks*, although from a statistical perspective there is one basic drawing task (see Fig. 2) performed with 2 (eyes open, eyes closed) × 2 (dominant hand, non-dominant hand) = 4 conditions. Data were analysed by a 2 × 2 × 2 × 3 mixed ANOVAs design with the within-subject factors time (pre, post), eye (open, closed) and hand (dominant, non-dominant), the between-subjects’ factor treatment-group (MPH, PHY, PP). Separate mixed ANOVAs were run for fluency, velocity and pen pressure. Post hoc tests for pairwise comparisons using Bonferroni correction were run. Since no equality of covariance was given for fluency (Box’s test *p* = 0.002), robust mixed ANOVAs (with 20% trimmed means) were run. As for fluency (in contrast to velocity and pen pressure) no 4-factor design could be run within a robust mixed ANOVA, since the WRS package is limited to a maximum of 3 factors, we used a 3-factor design with the within-subject factors time (pre, post) and condition (eyes open-dominant hand, eyes closed-dominant hand, eyes open-non-dominant hand, eyes closed-non-dominant hand), and the between-subjects’ factor treatment-group (MPH, PHY, PP). Further analysis by robust three-way ANOVA was run to analyse the conditions eye and hand as separated factors. Separate repeated measures (rm) ANOVAs for each treatment-group with time, eye and hand as within factors were run to confirm interactions with time for each treatment-group. To test whether the null hypothesis (H0) is more likely than the alternative hypothesis (H1) for the interaction of time  ×  treatment group, we additionally performed Bayesian analyses with time as within and treatment-group as between factor. According to Raftery [36] p (H_0_|D) values of 50–75% can be regarded as weak evidence for the null hypothesis, values of 75–95% can be regarded as positive evidence for the null hypothesis, values of 95–99% can be regarded as strong evidence for the null hypothesis. Furthermore, sensitivity analyses were run for the interaction of time  ×  treatment group. This analysis provides the effect size that is required to detect an effect at an *α* error probability of 5% and a power of 80% in the given sample. The calculated sensitivity is *f* = 0.43, meaning that the interaction of time x treatment group could be reliably detected if it explains ≥ 15.65% of the variance. Mixed ANOVAs and rm ANOVAS were run in SPSS (Version 28.0.1.0). Robust ANOVAs, were run in R (RStudio 2021.09.2-382, package WRS). Bayesian analyses were run in JASP (Version 0.16.3) and sensitivity analyses were run in G-Power (Version 3.1.9.4).

## Results

There was no difference between the three groups in sex and motor skills (Pearson’s chi-squared tests) as well as for age and the means of fluency, velocity and pen pressure (Kruskal Wallis tests, see Table 1).

**Table 1:** Descriptives for the three treatment groups

	Methylphenidate treatment group (*N* = 16)	Physiotherapeutic treatment group (*N* = 17)	Parental psycho-education group (*N* = 22)	Test statistic for overall group comparison
Sex (male/female)	14/2	15/2	17/5	*X*^2^ (2) = 1.00, *p* = 0.66
Below average motor skills, *n* (*%)*	9 (56.25)	15 (88.24)	13 (59.09)	*X*^2^ (2) = 5.12, *p* = 0.08
Age, M (SD)	9.56 (1.86)	9.29 (1.65)	9.95 (2.10)	*X*^2^ (2) = 0.90 *p* = 0.64
Fluency^a^, *M* (SD)				
Pre	2.87 (1.17)	2.61 (1.18)	2.33 (0.87)	*X*^2^ (2) = 2.33, *p* = 0.31
Post	2.54 (1.20)	2.27 (0.70)	2.14 (0.63)	*X*^2^ (2) = 0.46, *p* = 0.80
Velocity^b^, *M* (SD)				
Pre	1.76 (0.40)	1.95 (0.52)	1.95 (0.43)	*X*^2^ (2) = 2.37, *p* = 0.31
Post	1.99 (0.56)	2.11 (0.41)	2.14 (0.44)	*X*^2^ (2) = 0.46, *p* = 0.80
Pen Pressure^c^, *M* (SD)				
Pre	2.09 (0.36)	1.96 (0.52)	2.25 (0.44)	*X*^2^ (2) = 3.61, *p* = 0.17
Post	2.22 (0.40)	1.96 (0.62)	2.20 (0.49)	*X*^2^ (2) = 2.22, *p* = 0.33

Untransformed values; ^a^Mean fluency above task 1–6 measured by Number of Inversions in Velocity per stroke; ^b^Mean velocity above task 1–6 measured in Hz; ^c^Mean pressure above task 1–6 measured in Newton

### Kinematic parameters in everyday handwriting

Mixed ANOVA of *fluency* revealed a main effect of task [*F*(1, 52) = 8.29, *p* ≤ 0.01, *η*^2^ = 0.14] and an interaction of task  ×  treatment-group [*F*(2, 52) = 3.87, *p* ≤ 0.05, *η*^2^ = 0.13]. Post-hoc tests by pairwise comparison of estimated marginal means revealed higher NIV values (indicating lower fluency) in writing double-loops [*M* = 0.77, SE = 0.08] compared to writing a sentence [*M* = 0.61, SE = 0.08, *p* ≤ 0.01, corrected for three comparisons] only for the PHY group [MPH: double-loops *M* = 0.77, SE = 0.08, sentence *M* = 0.69, SE = 0.08, *p* = 0.12; PP: double-loops *M* = 0.67, SE = 0.07; sentence *M* = 0.68, SE = 0.07, *p* = 0.79].

Analysis of *velocity* revealed no interaction effects. Neither a 3-factor interaction (time  ×  task  ×  treatment-group) nor a 2-factor interaction (time  ×  task; time  ×  treatment-group; task  ×  treatment-group) was found [*F* = 0.75–2.68, *p* = 0.08–0.40, *η*^2^ = 0.01–0.09]. Furthermore, no main effect of time [*F*(1,52) = 3.33, *p* = 0.07, *η*^2^ = 0.06] or treatment-group [*F*(2,52) = 0.36, *p* = 0.70, *η*^2^ = 0.01] was found. A main effect of task [*F*(1, 52) = 4.13, *p* ≤ 0.05, *η*^2^ = 0.07] revealed slightly higher *velocity* in writing a sentence [*M* = 1.25, SD = 0.22] compared to writing double-loops [*M* = 1.21, SD = 0.20] across the treatment-groups.

No effects were found for *pen pressure* [*F* = 0.05–1.91, *p* = 0.16–0.94, *η*^2^ = 0.001–0.07]. That there is no interaction of time  ×  treatment-group for the three kinematic parameters is corroborated by the Bayesian analysis (see Table 2).

**Table 2:** Likelihood of the H0 for interactions of time x treatment-group

Measure	Tasks	p(H0|D), %	Evidence for H0
Fluency	Everyday handwriting tasks	99.3	Strong evidence
	Basic drawing	86.5	Positive evidence
Velocity	Everyday handwriting tasks	91.4	Positive evidence
	Basic drawing	100	Strong evidence
Pressure	Everyday handwriting tasks	97.2	Strong evidence
	Basic drawing	96.3	Strong evidence

### Kinematic parameters in basic drawing

Robust mixed ANOVA of *fluency* revealed a main effect of time [*F*(1,52) = 3.99, *p* ≤ 0.05, *η*^2^ = 0.07] with lower values (indicating increased fluency) after treatment [*M* = 0.36, SD = 0.19] compared to values before treatment [*M* = 0.42, SD = 0.20] across all treatment-groups and a main effect of condition [*F*(3,156) = 4.83, *p* ≤ 0.01, *η*^2^ = 0.08].

**Fig. 3:**
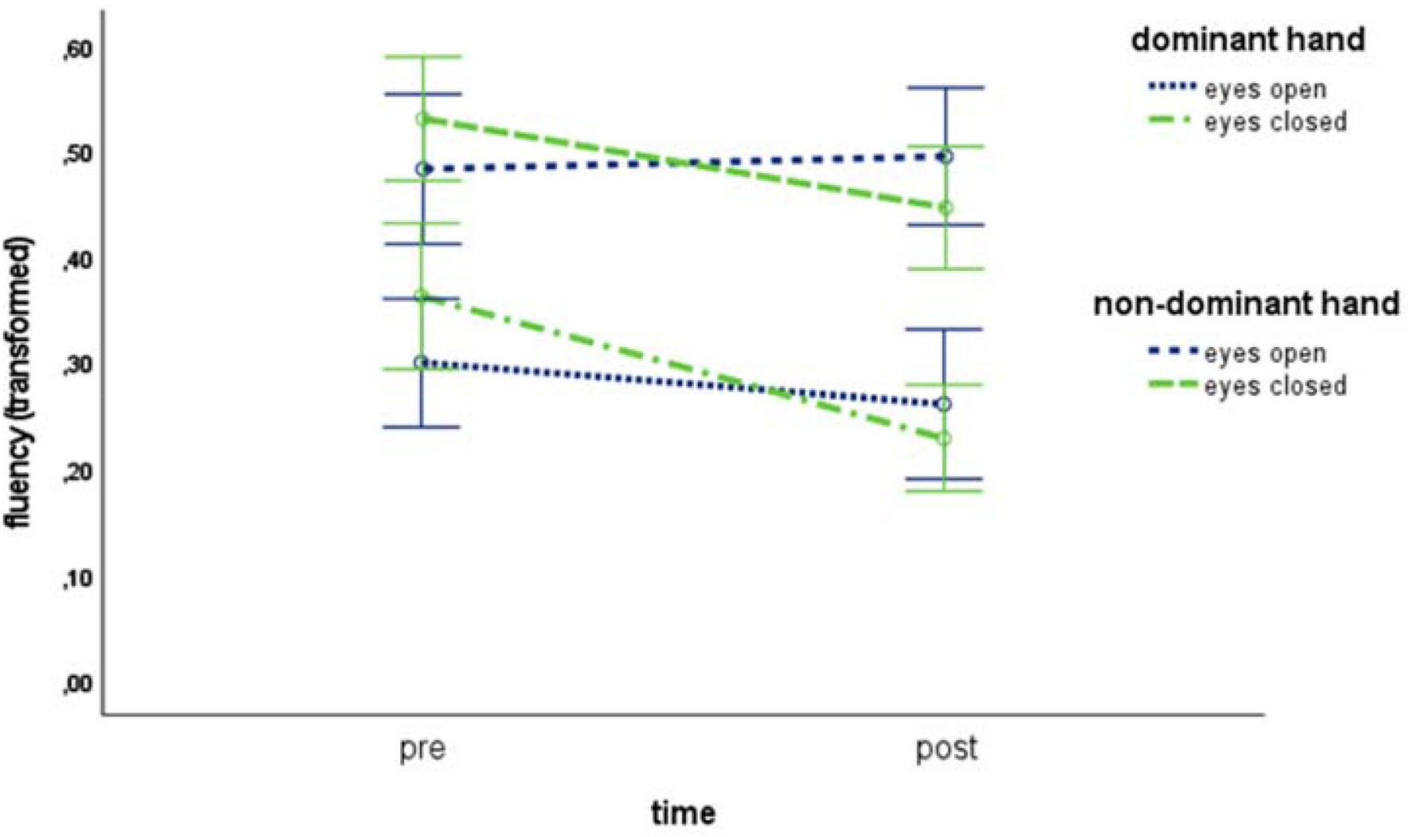
Effects of the factors time and eye for the dominant and non-dominant hand on GM fluency in basic drawing tasks of all three treatment-groups together with 95% CI

A further analysis by robust three-way ANOVA with the within factors time, eye and hand was run. Results, visualized in Fig. 3, revealed an interaction of time  ×  eye [*F*(1,54) = 9.32, *p* ≤ 0.01, *η*^2^ = 0.15] and time  ×  hand [*F*(1,54) = 6.61, *p* ≤ 0.05, *η*^2^ = 0.11] as well as main effects of time [*F*(1,54) = 4.45, *p* ≤ 0.05, *η*^2^ = 0.08] and hand [*F*(1,54) = 67.67, *p* ≤ 0.01, *η*^2^ = 0.56]. The NIV values decreased (indicating increasing fluency) after treatment when the task was performed with the eyes closed [20% trimmed, pre: *M* = 0.44, SD = 0.29, post: *M* = 0.32, SD = 0.27, *p* ≤ 0.01], while they remained more stable during the performance of the task with the eyes open [20% trimmed, pre: *M* = 0.38, SD = 0.31, post: *M* = 0.36, SD = 0.36, *p* = 0.67]. Moreover, NIV values decreased (indicating increasing fluency) after treatment when the task was performed with the dominant hand [20% trimmed, pre: *M* = 0.30, SD = 0.26, post: *M* = 0.20, SD = 0.20, *p* ≤ 0.01], while they remained more stable during the performance of the task with the non-dominant hand [20% trimmed, pre: *M* = 0.52, SD = 0.27, post: *M* = 0.49, SD = 0.26, *p* = 0.55].

Analysis by separate rm ANOVAS for each treatment-group with time, eye and hand as the within-subjects factors confirmed the interaction of time  ×  eye for the MPH and PHY group [MPH: *F*(1,15) = 11.00, *p* ≤ 0.01, *η*^2^ = 0.42; PHY: *F*(1,16) = 4.64, *p* ≤ 0.05, *η*^2^ = 0.23] with decreased NIV values (indicating increasing fluency) after treatment as before treatment in tasks with eyes closed. Higher NIV values (indicating lower fluency) were found in tasks performed with eyes closed compared to tasks performed with eyes open before treatment within MPH group (for means and standard deviations refer to Tables 1–3 of supplementary material). The interaction of time  ×  hand was only confirmed for the PP group [*F*(1,21) = 9.31, *p* ≤ 0.01, *η*^2^ = 0.31] with decreased NIV values (indicating increasing fluency) after treatment in the dominant hand (for means and standard deviations refers to Tables 1–3 of supplement material).

**Fig. 4:**
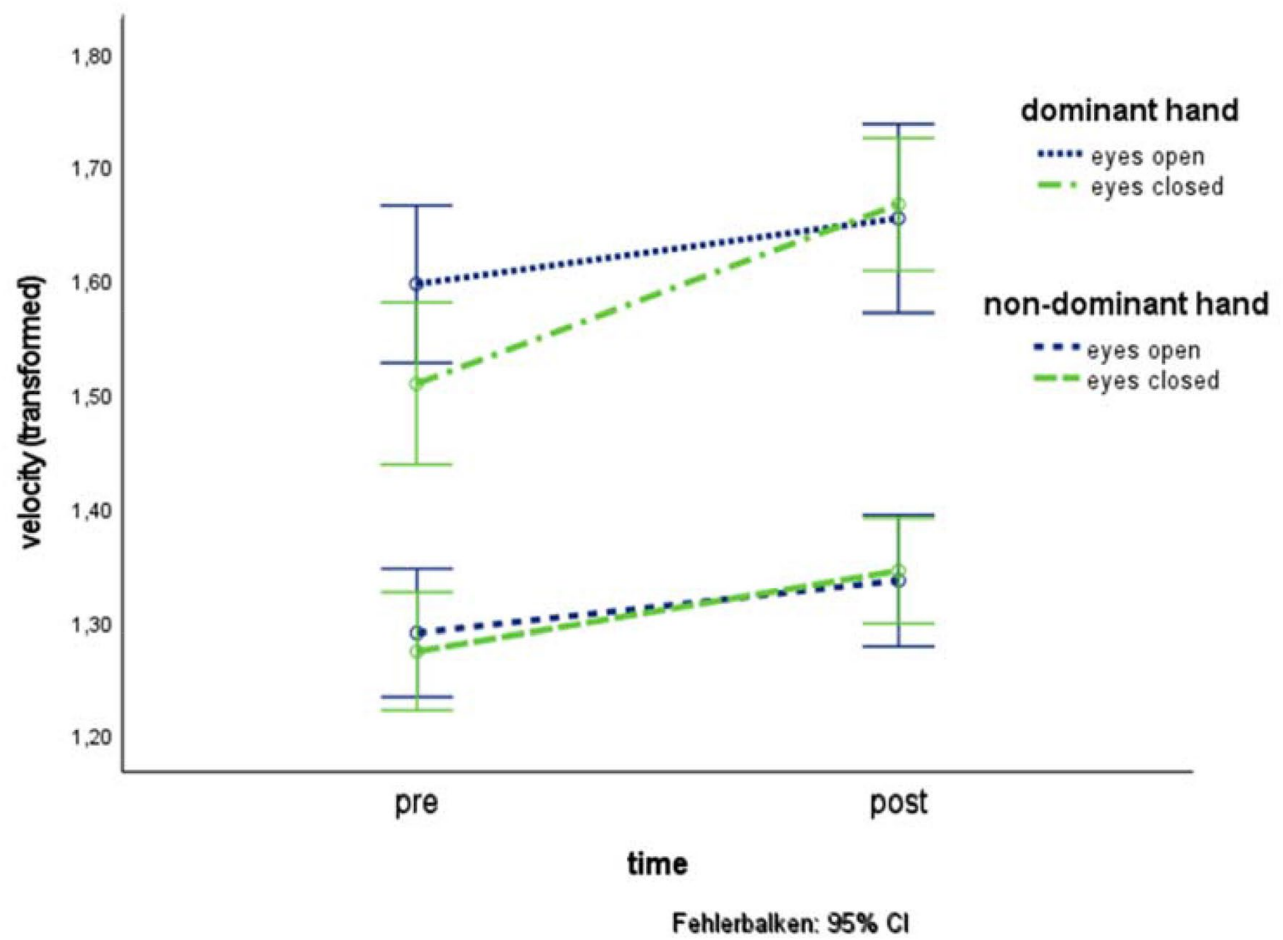
Effects of the factors time and eye for the dominant and non-dominant hand on GM velocity in basic drawing tasks of all three treatment-groups together with 95% CI

For *velocity*, a main effect of time [*F*(1, 52) = 9.47, *p* ≤ 0.01, *η*^2^ = 0.15] with higher velocity after treatment [*M* = 1.50, SD = 0.20] as before treatment [*M* = 1.41, SD = 0.19] across all treatment-groups and main effect of hand [*F*(1, 52) = 148.39, *p* ≤ 0.01, *η*^2^ = 0.74] with higher velocity of the dominant hand [*M* = 1.61, SD = 0.21] compared to the non-dominant hand [*M* = 1.31, SD = 0.16] was found. Furthermore, an interaction effect of time  ×  eye [*F*(1, 52) = 9.28, *p* ≤ 0.01, *η*^2^ = 0.15] was found (see Fig. 4). Post hoc tests by pairwise comparison of estimated marginal means revealed higher velocity after treatment in tasks performed with eyes closed [*M* = 1.50, SE = 0.24] as before treatment [*M* = 1.39, SE = 0.3, *p* ≤ 0.01, corrected for two comparisons] and higher velocity in tasks performed with eyes open [*M* = 1.44, SE = 0.03] compared to tasks performed with eyes closed [*M* = 1.39, SE = 0.03] before treatment (*p* ≤ 0.01, corrected for two comparisons).

Separate rm ANOVAs for each treatment-group with time, eye and hand as within-subject factors were run. The main effect of hand was confirmed for all three groups [MPH: *F*(1, 15) = 18.69, *p* ≤ 0.01, *η*^2^ = 0.56; PHY: *F*(1, 16) = 67.47, *p* ≤ 0.01, *η*^2^ = 0.81; PP: *F*(1,21) = 88.02, *p* ≤ 0.01, *η*^2^ = 0.81]. The interaction of time  ×  eye was only significant in the MPH group [*F*(1, 15) = 4.61, *p* ≤ 0.05, *η*^2^ = 0.24] with increased velocity after treatment in tasks with eyes closed. For the PP group an interaction eye  ×  hand was found [*F*(1, 21) = 6.87, *p* ≤ 0.05, *η*^2^ = 0.25] with higher velocity in the dominant hand in tasks performed with eyes open, while this was reverse in the non-dominant hand. Furthermore an interaction time  ×  hand [*F*(1, 21) = 5.07, *p* ≤ 0.05, *η*^2^ = 0.20] with increased velocity after treatment in the dominant hand was found in the PP group.

For *pen pressure* a large main effect of hand [*F*(1, 52) = 140.60, *p* ≤ 0.01, *η*^2^ = 0.73] with higher pressure in the dominant hand [*M* = 1.51, SD = 0.18] compared to the non-dominant hand [*M* = 1.25, SD = 0.16] across all treatment-groups was found. Furthermore, there was an interaction for hand  ×  treatment-group [*F*(2, 52) = 4.18, *p* ≤ 0.05, *η*^2^ = 0.14] and a 3-factor interaction effect for eye  ×  hand  ×  treatment-group [*F*(2, 52) = 3.46, *p* ≤ 0.05, *η*^2^ = 0.12]. Post hoc tests by pairwise comparison of estimated marginal means confirmed higher pressure in the dominant hand compared to the non-dominant hand for each treatment-group (*p* ≤ 0.01 for each treatment-group, corrected for three comparisons). Furthermore, post hoc tests by pairwise comparison of estimated marginal means reveals for the PP group lower pen pressure in the dominant hand in tasks performed with eyes closed [*M* = 1.54, SE = 0.04] compared to tasks performed with eyes open [*M* = 1.61, SE = 0.04, *p* ≤ 0.01, corrected for six comparisons].

That there is no interaction of time  ×  treatment-group for the three kinematic parameters is corroborated by the Bayesian analysis (see Table 2).

## Discussion

The current study investigated the impact of two different treatments over eight weeks (methylphenidate or physiotherapeutic treatment) compared to a control treatment (parental psychoeducation) on GM in children with ADHD. Six different tasks were performed on a digitizing tablet which allowed the analysis of three objectively measured and important parameters of GM: fluency, velocity, and pen pressure.

The results of the present clinical sample indicate that GM neither at the end of week 8 of methylphenidate treatment nor after 8 weeks of physiotherapeutic treatment improve more than after the control condition parental psychoeducation (an overview of the treatment effects is shown in Fig. 1 in the supplementary materials). The results of the analysis of variance were corroborated by the Bayesian statistics, with positive to strong confirmation of the null hypotheses regarding the interactions between time and treatment-group. Looking at the treatment groups separately, effects were not as hypothesized. Contrary to our assumption of writing and drawing less fluently, slower and with a higher pen pressure when treated with methylphenidate in tasks performed with eyes open, we found no worsening or other change of fluency, velocity and pen pressure. Also contrary to our hypothesis there was an improvement of fluency and velocity in tasks performed with eyes closed when treated with methylphenidate. As hypothesized we found no worsening or improvement of pen pressure in tasks with eyes closed when treated with methylphenidate.

Also for physiotherapeutic treatment, the hypothesized treatment effects of more fluency, lower velocity and less pen pressure in all tasks were not found. There was no change in the three GM parameters after physiotherapeutic treatment in tasks performed with eyes open, while there was an improvement in tasks performed with eyes closed. Still, the observed improvement was less pronounced than for the methylphenidate treatment.

Contrary to our expectations, we found treatment effects for parental psychoeducation. Children whose parents participated in parental psychoeducation showed improved fluency and velocity in the dominant hand after this control treatment. This result is surprising as psychoeducation is not a specific intervention on motor skills or attention, two factors that we expect to have a positive impact on GM, and raises the question of which factors of psychoeducation have led to an improvement in fluency and velocity. There are some potential factors that may have had a positive impact on graphomotor skills. For example, adapting parenting methods to children’s symptoms can lead to a more structured daily routine, giving parents more resources to support their children, for example through motor activities. In addition, a more conscious handling of the children’s difficulties by the parents could lead to an increased self-confidence of the children, which in turn could have a positive effect on motor performance. A reduction in stress levels in the children (due to the change in the way parents deal with the children’s difficulties) could also have an effect on motor performance, as glucocorticoid receptors are present in the motor system and therefore stress hormones may influence motor system function. There is another study that indicates an improvement in motor skills after parental psychoeducation. Comparing a training on executive, attention, and motor skills in preschool children with a parent education and support program reveals improvements in manual motor skills irrespective of treatment condition [37]. Further studies are needed to understand the effect of parental psychoeducation on children’s motor functions. However, there was no specific improvement in tasks performed with eyes closed, as with methylphenidate or after physiotherapeutic treatment. No treatment effects were found for pen pressure, in any of the tasks for any of the three groups.

The expected treatment-unspecific effects between the dominant and non-dominant hand could be confirmed (more fluency, velocity and pen pressure before and after treatment in the dominant hand compared to the non-dominant hand across all groups).

In general, the results of the fluency and velocity measures indicate more robust improvements over time in GM without visual feedback than in GM with visual feedback, across all participants. This is in line with previous results demonstrating that children with ADHD struggle with the integration of constant visual feedback and retrieval of learned motor programs [38, 39]. However, it does not explain why we do not find the expected worsening in fluency and velocity on methylphenidate in tasks with visual feedback shown by others [20, 21]. One reason for this could be that we studied the effects at the end of week 8 of methylphenidate treatment compared to the previous methylphenidate-naive state and not during ongoing methylphenidate treatment compared to withdrawal from methylphenidate (10–12 h after the last medication) as in the studies of Tucha and Lange [20, 21]. When withdrawing methylphenidate, complex tasks such as writing sentences that require visual-motor integration [38, 39] and concurrent retrieval of spellings from the mental orthographic lexicon [40] might be overloading and lead to an enhanced focus on the primary task, in the present study GM, with improved outcome. On the other hand, in the present study, the beneficial effect of methylphenidate on GM with visual feedback may not occur soon after starting treatment, as GM without visual feedback need to be more automatized first to improve tasks with visual motor integration. This can also be assumed for the physiotherapeutic treatment.

### Strengths and limitations

A strength of the present study is that it was set in routine clinical practice to investigate the effects of routinely administered physical exercise on fine motor skills in children with ADHD, which has not been studied in this way before. Therefore, the observed effects give indications of treatment effects that might be achieved in the scope of clinical treatments. Another strength of the study is the utilization of a digitizing tablet to collect objective quantitative data on several parameters of GM in children with ADHD.

In addition to the number of participants, there are other limiting factors of our study. First, no randomized design was used for the GM tasks. Second, within the group of physiotherapeutic treatment most of participants (88%) show below average motor skills during the diagnostic process, while in the MPH and parental psychoeducation group just over half of participants show below average motor skills. This limits the comparison of effects between groups, as advances are achieved more slowly in the more affected group (physiotherapeutic treatment) and thus the effects after 8 weeks could be smaller than in a less affected group. Third, it must be noted that some participants of the physiotherapeutic treatment (*n* = 4) and parental psychoeducation (*n* = 9) group took methylphenidate before and while taking part in the study, but the medication was not changed during the study. The influence of methylphenidate was therefore the same before and after physiotherapeutic treatment or parental psychoeducation, and the changes in GM could not be an effect of methylphenidate. However, the intervention effects in these participants could be biased such that methylphenidate already reinforced GM and the effects could not be achieved by an additional intervention due to ceiling effects.

## Conclusion

In summary, we present objective data on GM in children with ADHD under different treatment conditions. We were not able to show clear evidence that children’s overall GM benefited from 8 week of treatment using methylphenidate or physiotherapeutic treatment more than from parental psychoeducation (= control condition). However, the present results demonstrate that graphomotor skills of children with ADHD improved most in basic drawing movements without visual feedback, across all participants. It can be assumed that the benefit from improved attentional performance is greatest in these stimulus-reduced tasks. How improved attentional performance in children with ADHD could lead to improved visual-motor integration (movements with visual feedback) should be investigated in future studies. In clinical practice, fine motor skills including GM should be given more consideration of prescribing and treating with physiotherapy.

### Supplementary Information

Below is the link to the electronic supplementary material.

Supplementary file1 (PDF 959 KB)

## Data Availability

The datasets used during the current study are available from the corresponding author on reasonable request.
