# Supplementary material for: Effects of methylphenidate and physiotherapeutic treatment on graphomotor movements in children with ADHD
Source: Eur Child Adolesc Psychiatry. 2023 Jan 23;33(1):127–37. doi: 10.1007/s00787-023-02144-5 (PMC10806214; doi:10.1007/s00787-023-02144-5)

Supplementary Material

Table 1S. Means and standard deviation (in parentheses) of transformed (λ = -.90) handwriting fluency (NIV) of each tasks for the three treatment-groups

	MPH (N = 16)		PHY (N = 17)		PP (N = 22)	
	pre	post	pre	post	pre	post
everyday handwriting tasks						
sentence	.56	.59	.52	.49	.55	.57
	(.22)	(.20)	(.27)	(.27)	(.24)	(.23)
double-loops	.62	.61	.62	.64	.55	.55
	(.27)	(.26)	(.23)	(.22)	(.28)	(.22)
basic drawing tasks						
dominant hand	.39	.38	.28	.25	.25	.18
eyes open	(.26)	(.33)	(.23)	(.26)	(.18)	(.24)
non-dominant	.50	.44	.47	.52	.48	.51
hand eyes open	(.27)	(.28)	(.30)	(.23)	(.25)	(.22)
dominant hand	.49	.27	.33	.24	.30	.19
eyes closed	(.24)	(.21)	(.26)	(.20)	(.19)	(.14)
non-dominant	.60	.39	.55	.46	.46	.47
hand eyes closed	(.19)	(.24)	(.25)	(.19)	(.16)	(.22)

Table 2S. Means and standard deviation (in parentheses) of transformed (λ = .50) handwriting velocity (Hz) of each tasks for the three treatment-groups

	MPH (N = 16)		PHY (N = 17)		PP (N = 22)	
	pre	post	pre	post	pre	post
everyday handwriting tasks						
sentence	1.25	1.21	1.26	1.33	1.22	1.24
	(.21)	(.22)	(.27)	(.24)	(.22)	(.24)
double-loops	1.18	1.17	1.21	1.25	1.20	1.27
	(.26)	(.25)	(.20)	(.22)	(.18)	(.19)
basic drawing tasks						
dominant hand	1.49	1.54	1.63	1.68	1.65	1.73
eyes open	(.25)	(.41)	(.28)	(.28)	(.23)	(.23)
non-dominant	1.27	1.36	1.3	1.32	1.28	1.33
hand eyes open	(.22)	(.27)	(.22)	(.16)	(.20)	(.21)
dominant hand	1.40	1.62	1.54	1.66	1.57	1.71
eyes closed	(.25)	(.26)	(.27)	(.20)	(.26)	(.20)
non-dominant	1.22	1.35	1.26	1.34	1.32	1.34
hand eyes closed	(.20)	(.18)	(.20)	(.12)	(.17)	(.20)

Table 3S. Means and standard deviation (in parentheses) of transformed (λ = .50) handwriting pressure (Newton) of each tasks for the three treatment-groups

	MPH (N = 16)		PHY (N = 17)		PP (N = 22)	
	pre	post	pre	post	pre	post
everyday handwriting tasks						
sentence	1.52	1.53	1.48	1.51	1.59	1.60
	(.21)	(.14)	(.27)	(.27)	(.18)	(.21)
double-loops	1.53	1.56	1.46	1.44	1.60	1.59
	(.15)	(.15)	(.28)	(.29)	(.21)	(.20)
basic drawing tasks						
dominant hand	1.48	1.51	1.45	1.47	1.65	1.57
eyes open	(.17)	(.17)	(.22)	(.26)	(.21)	(.23)
non-dominant	1.29	1.35	1.24	1.22	1.26	1.22
hand eyes open	(.19)	(.21)	(.22)	(.19)	(.17)	(.19)
dominant hand	1.51	1.54	1.42	1.41	1.54	1.54
eyes closed	(.17)	(.16)	(22)	(.28)	(.20)	(.21)
non-dominant	1.25	1.37	1.21	1.18	1.23	1.26
hand eyes closed	(.16)	(.25)	(.19)	(.17)	(.20)	(.20)

Figure 1S. Interaction of time and treatment-group in measures of fluency, velocity and pen pressure across everyday handwriting tasks and basic drawing tasks

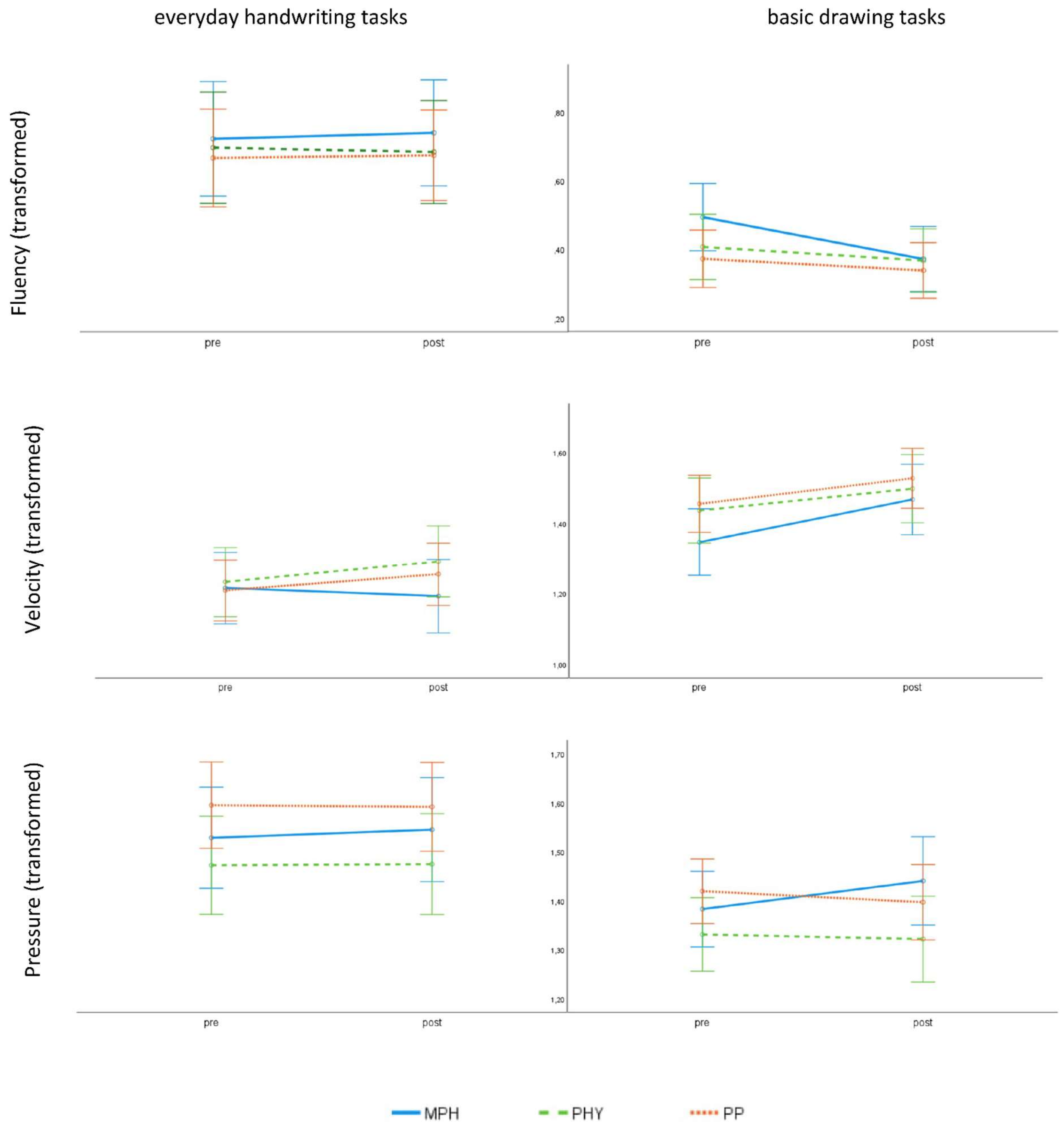

Figure 2S. Boxplots of untransformed fluency measures in basic drawing tasks

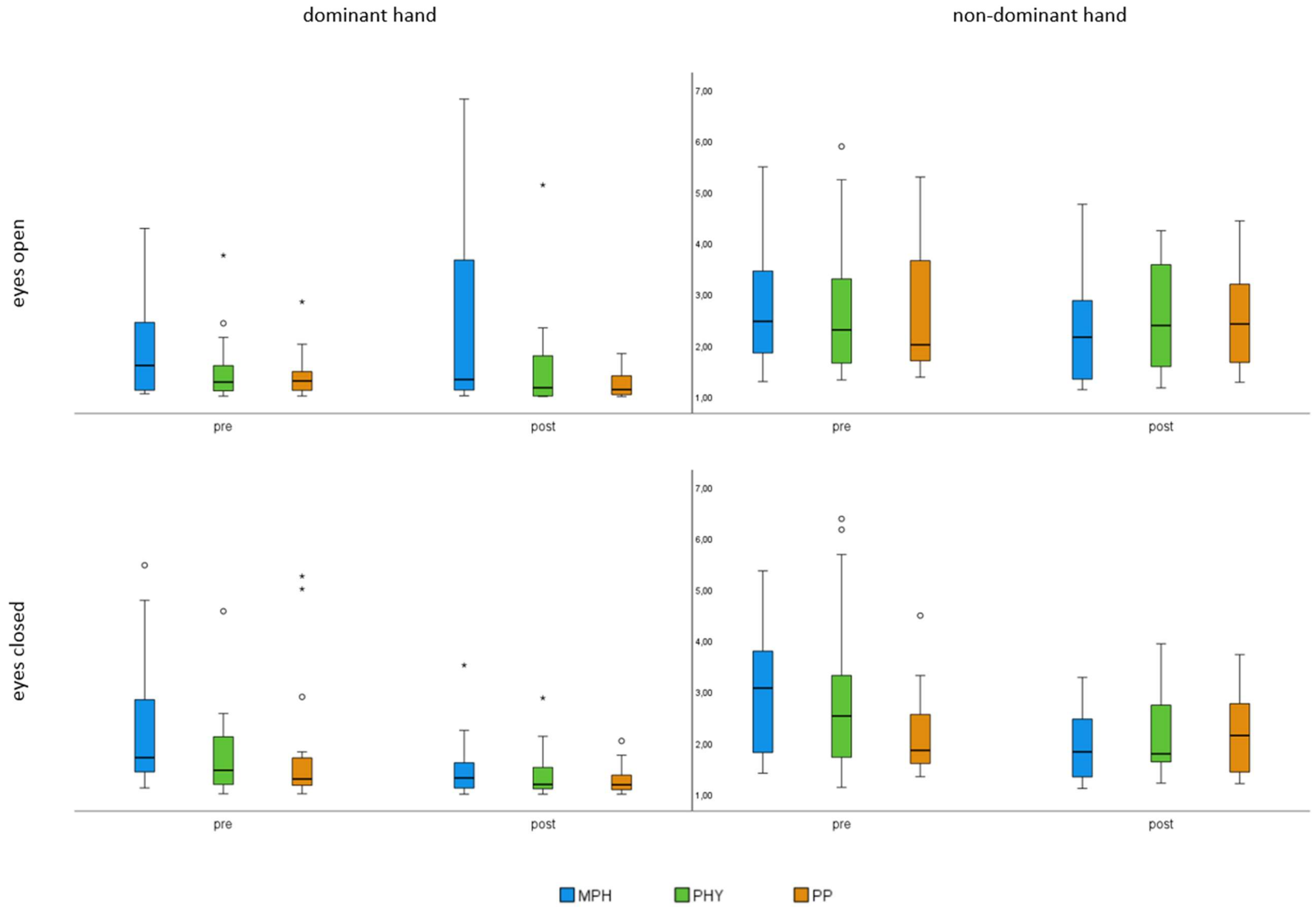

Figure 3S. Boxplots of untransformed velocity measures in basic drawing tasks

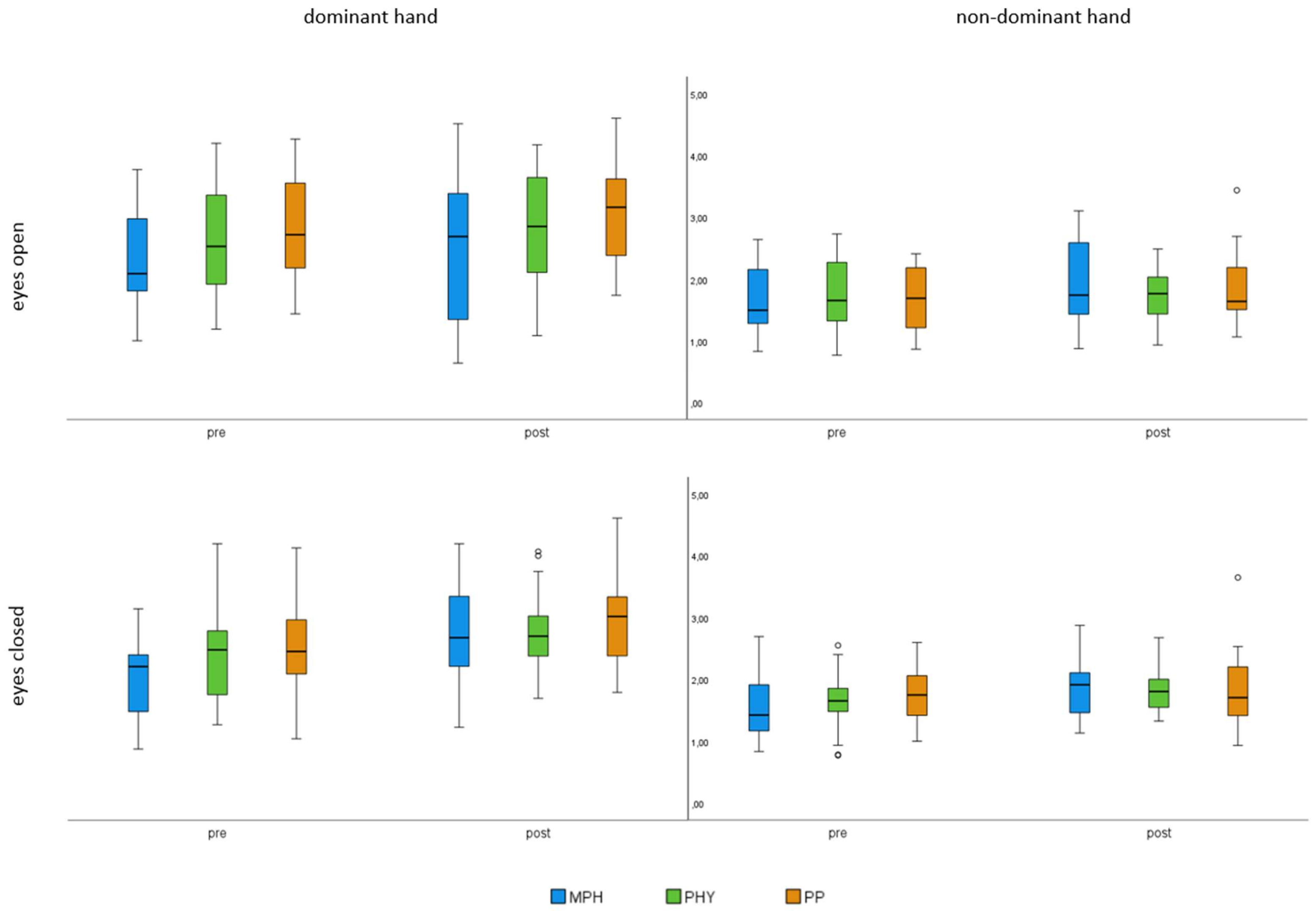

Figure 4S. Boxplots of untransformed pressure measures in basic drawing tasks

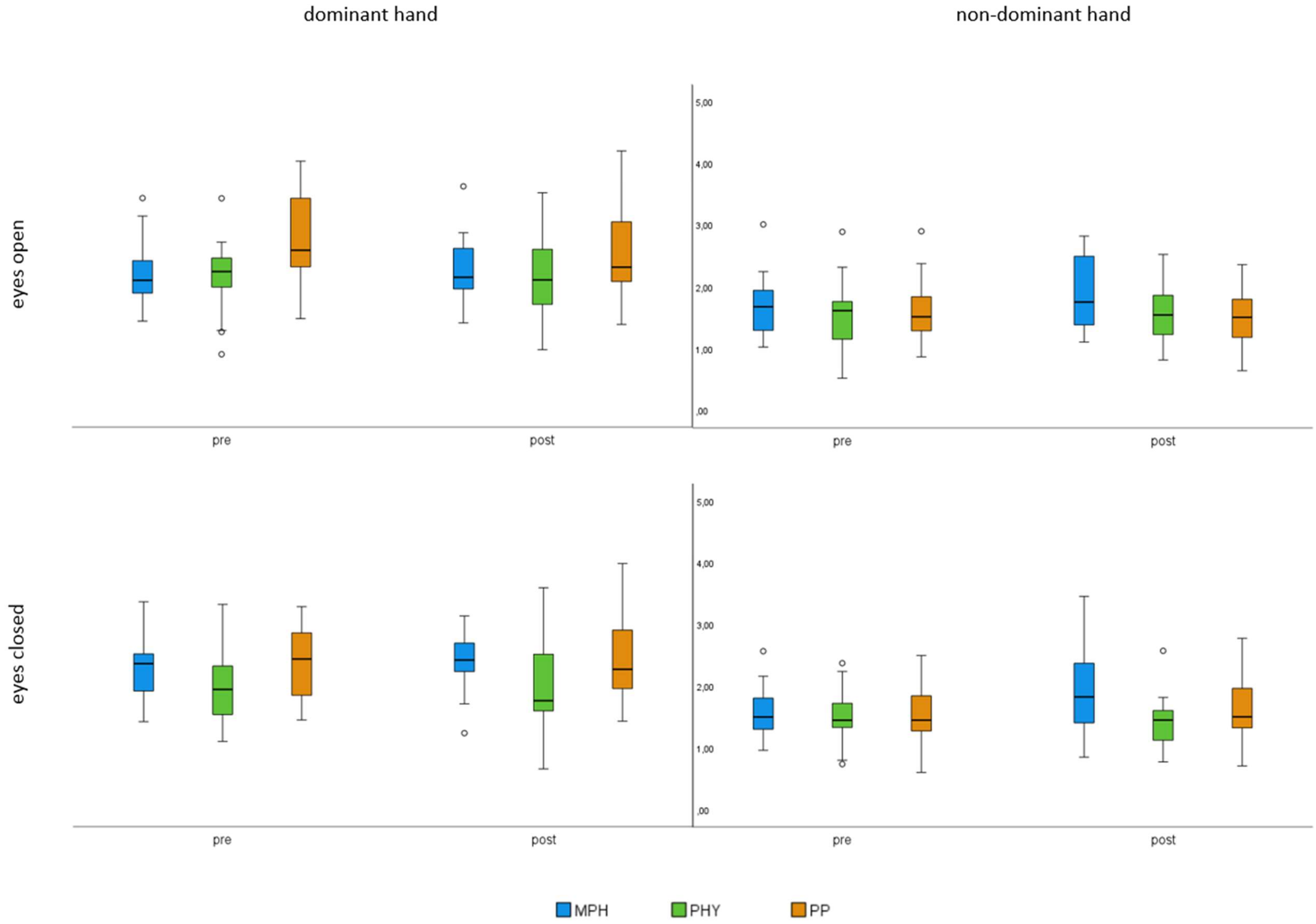

Supplement: Supplementary file 1 — Supplementary file1 (PDF 959 KB) [file 787_2023_2144_MOESM1_ESM.pdf]
